# Integrated bioinformatic analysis of gene expression profiling data to identify combinatorial biomarkers in inflammatory skin disease

**DOI:** 10.1038/s41598-022-09840-3

**Published:** 2022-04-07

**Authors:** Heejin Bang, Ja Eun Kim, Hyun Su Lee, Sang Man Park, Dong-Joon Park, Eun Jung Lee

**Affiliations:** 1 Department of Pathology, Konkuk University Medical Center, Konkuk University School of Medicine, Seoul, Republic of Korea; 2 Yonsei University Wonju College of Medicine, Wonju, Republic of Korea; 3 Department of Otorhinolaryngology, Yonsei University Wonju College of Medicine, Wonju, Republic of Korea

**Keywords:** Computational biology and bioinformatics, Systems biology, Biomarkers, Medical research

## Abstract

Selection of appropriate biomarker to identify inflammatory skin diseases is complicated by the involvement of thousands of differentially expressed genes (DEGs) across multiple cell types and organs. This study aimed to identify combinatorial biomarkers in inflammatory skin diseases. From one gene expression microarray profiling dataset, we performed bioinformatic analyses on dataset from lesional skin biopsies of patients with inflammatory skin diseases (atopic dermatitis [AD], contact eczema [KE], lichen planus [Li], psoriasis vulgaris [Pso]) and healthy controls to identify the involved pathways, predict upstream regulators, and potential measurable extracellular biomarkers. Overall, 434, 629, 581, and 738 DEGs were mapped in AD, KE, Li, and Pso, respectively; 238 identified DEGs were shared among four different inflammatory skin diseases. Bioinformatic analysis on four inflammatory skin diseases showed significant activation of pathways with known pathogenic relevance. Common upstream regulators, with upregulated predicted activity, identified were *CNR1* and *BMP4*. We found the following common serum biomarkers: ACR, APOE, ASIP, CRISP1, DKK1, IL12B, IL9, MANF, MDK, NRTN, PCSK5, and VEGFC. Considerable differences of gene expression changes, involved pathways, upstream regulators, and biomarkers were found in different inflammatory skin diseases. Integrated bioinformatic analysis identified 12 potential common biomarkers of inflammatory skin diseases requiring further evaluation.

## Introduction

Inflammatory skin diseases are complex, chronic, multifactorial disorders, which are characterized by activation of the innate and adaptive immune system via production of proinflammatory cytokines^1^. Environmental, genetic, and immunologic factors apparently play a role in the pathogenesis of inflammatory skin diseases^2^. Itchy, red, or unsightly inflammatory skin conditions, such as hives, eczema, or psoriasis, can affect the quality of life and may be associated with psychological distress for the patients. Furthermore, common chronic inflammatory skin diseases, including atopic dermatitis (AD), contact eczema (KE), lichen planus (Li), and psoriasis vulgaris (Pso), manifest a relapsing and remitting course throughout the patient’s life^1,2^.

The host skin-based defence system comprises a barrier, innate immunity, and acquired immunity. The pathophysiology of inflammatory skin diseases involves various inflammatory cells and the innate immune response^1^. A recent study suggested that epigenetic factors, a core subset of inflammation-associated differentially methylated genes, were crucially involved in the pathophysiology of inflammatory skin diseases^3^. As early diagnosis and monitoring are important to prevent disease progression, reliable biomarkers are needed. However, despite decades of painstaking research with genome-wide analyses, such measurable biomarkers have proven elusive. That is why current diagnostic strategies are predominantly based on clinic-pathological reports in combination with the clinical history and physical examination^4,5^. With bioinformatic research into the molecular network in the skin, targeted therapies and early therapeutic intervention have revolutionized management strategies in clinical practice^6^.

We hypothesized that novel and potentially more specific biomarkers for inflammatory skin disease could be identified through a bioinformatic analysis of existing profiling data obtained from skin tissues of patients with inflammatory skin diseases, in comparison to the data from healthy controls (HCs). Bioinformatical analysis, especially ingenuity pathway analysis (IPA), is focused on pathways and upstream regulators rather than individual genes and provides a functional overview of the complex gene expression changes in each dataset^7–9^. The analysis on pathways and upstream regulators suggested a considerable complexity causing difficulties in finding representative biomarkers. Therefore, we aimed to identify combinatorial biomarkers in inflammatory skin diseases.

## Results

### Selection of eligible microarray datasets

We searched the Gene Expression Omnibus (GEO) database using the terms ‘inflammatory skin disease, ‘human’, ‘healthy control’, and ‘microarray’. This resulted in the identification of one mRNA transcriptional profiling study (GSE 63741) of whole-skin biopsies from control subjects and diseased skin tissues from patients with inflammatory skin diseases, such as AD, KE, Li, and Pso, versus the HC^10^. This study performed an unsupervised cluster analysis of gene expression profiles in 30 Pso patients and other inflammatory skin diseases (30 AD, 30 Li, 30 KE, and 30 HC). The study was performed using the platform, PIQOR (TM) Skin 2.0 Microarray, human, antisense (591).

### Identification of differentially expressed genes in skin biopsies from patients with inflammatory skin disease

**Figure 1:**
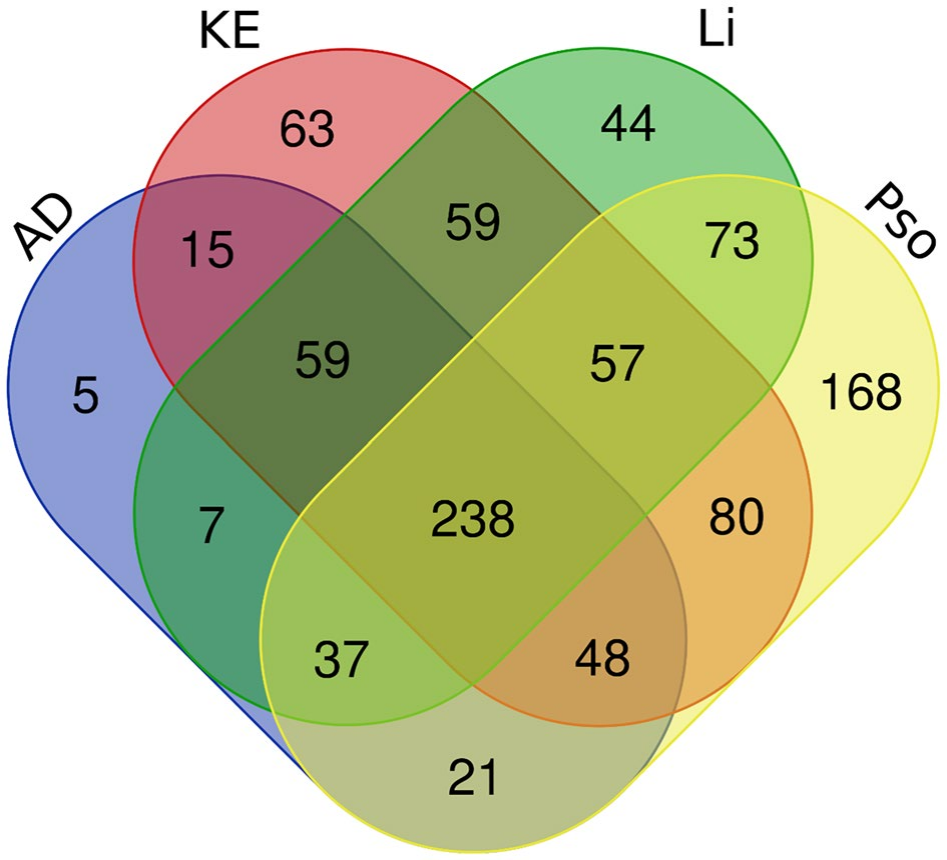
Venn diagram of differentially expressed genes in the inflammatory skin diseases. AD; atopic dermatitis, KE; contact eczema, Li; lichen planus, Pso; psoriasis vulgaris

We systematically analysed the datasets for differentially expressed genes (DEGs), shared pathways, predicted upstream regulators, and biomarkers. We initially identified the DEGs from the diseased skin samples of patients with inflammatory skin diseases versus the healthy skin samples from HC. We found that AD, KE, Li, and Pso had 434, 629, 581, and 738 DEGs, respectively (Supplementary Fig. 1, Supplementary Table 1); 238 DEGs overlapped among the four datasets (Fig. 1). These genes contained several known factors that are relevant for inflammatory skin diseases, including human leukocyte antigen genes, chemokines, cytokines, and T-cell immune-regulator genes, such as *TCIRG1*^11^. These analyses from both integration of gene ontology (GO) terms for DEGs and protein–protein interaction network supported the pathophysiological relevance of the shared 238 DEGs from the inflammatory skin lesions (Supplementary Fig. 2, Supplementary Table 2)^12–14^. Despite the presence of shared 238 DEGs, four different kinds of inflammatory skin disease represent different phenotypes. This led us to the current system-level analysis to examine whether there were pathway overlaps among the four different datasets.

### Identification of pathways in skin biopsy specimens from patients with inflammatory skin disease

**Figure 2:**
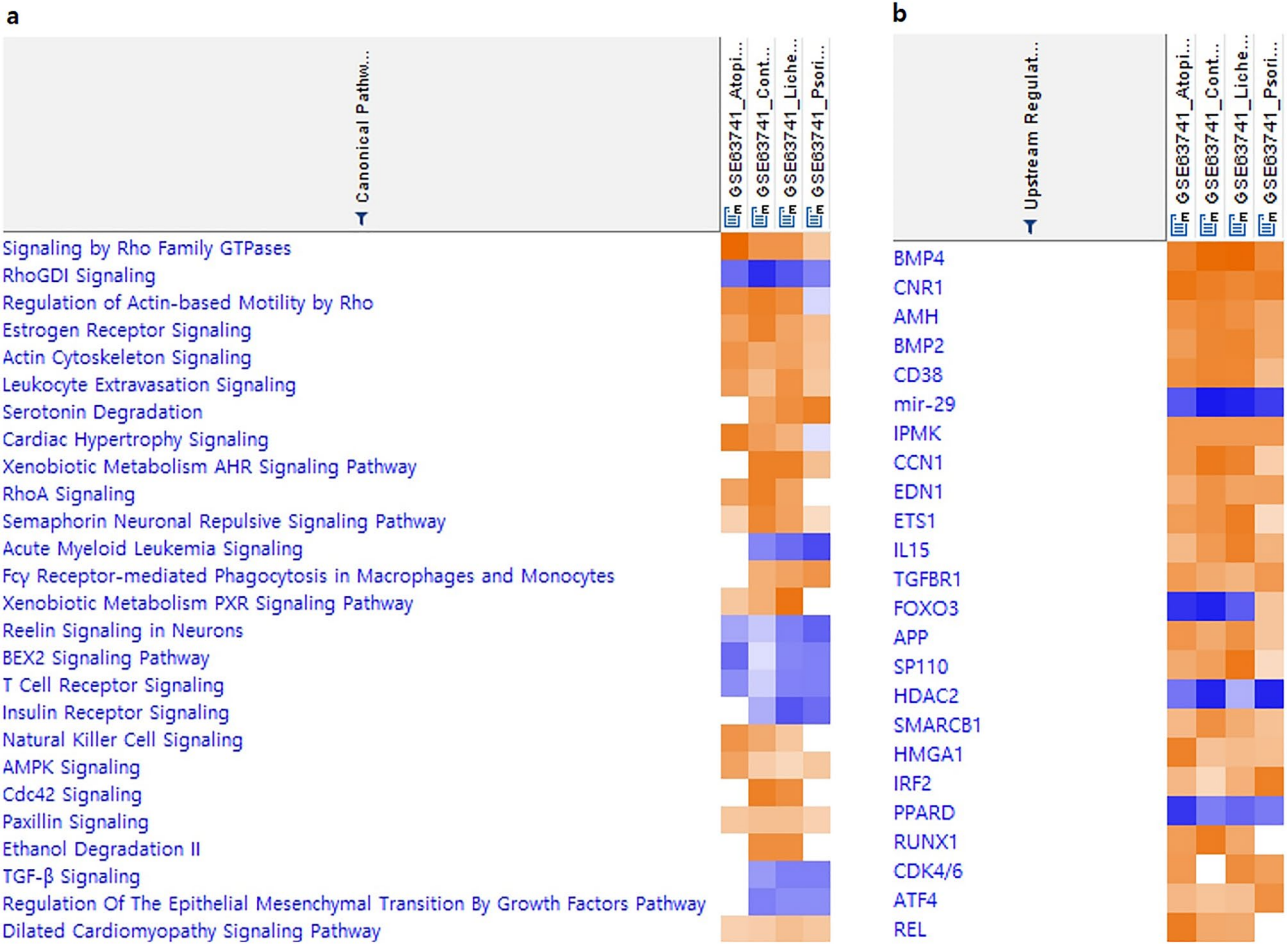
Comparison analysis (**a**) canonical pathways (CP), (**b**) upstream regulators (UR).

To obtain a functional overview of the complex gene expression changes in inflammatory skin diseases, we performed the IPA to identify disease-associated pathways. This resulted in the identification of a vast number of involved pathways for different inflammatory skin diseases (Supplementary Table 3). The top significant pathways (Z-score > 2) included signalling by Rho Family GTPases (2.646), white adipose tissue-browning pathway (2.236), xenobiotic metabolism general signalling pathway (2.236), cardiac hypertrophy signalling (2.236), regulation of actin-based motility by Rho (2), Tec kinase signalling (2) in AD, regulation of actin-based motility by Rho (2.236), xenobiotic metabolism aryl hydrocarbon receptor signalling pathway (2.236), Cdc42 signalling (2.236), RhoA signalling (2.236), estrogen receptor signalling (2.183), semaphorin neuronal repulsive signalling pathway (2.111), ethanol degradation II (2), agrin interactions at neuromuscular junction (2), STAT3 pathway (2) in KE, xenobiotic metabolism pregnane X receptor signalling pathway (2.449), xenobiotic metabolism AHR signalling pathway (2.236), ethanol degradation II (2), noradrenaline and adrenaline degradation (2), regulation of actin-based motility by Rho (2), Cdc42 signalling (2), serotonin degradation (2), synaptic long term potentiation (2) in LP, serotonin degradation (2.236), and synaptic long term depression (2) in Pso. Interestingly, comparative analysis of the four inflammatory skin diseases showed a similar pattern, which may explain the difficulty in clinically ascertaining the differential diagnosis in inflammatory skin diseases (Fig. 2a). These findings agreed with our previous findings that, despite the limited overlap of single genes between the gene expression profiling studies, there can be significant pathway overlap^15,16^. Collectively, the pathway analysis indicate a dynamic pathogenic complexity, which reflects a great challenge to the prioritization of biomarkers for inflammatory skin diseases.

### Identification of upstream regulators in inflammatory skin diseases

Next, we performed IPA analysis of the DEGs to identify the predicted activated upstream regulators of those DEGs from the diseased skin samples of patients with inflammatory skin diseases. The rationale for the analysis was that, similar to the pathways, such regulators constitute higher order representations of the complex gene expression changes (Supplementary Table 4). Paralleling with the pathways, the comparative analysis of upstream regulators showed a similar pattern among the four different inflammatory diseases (Fig. 2b). We listed 5, 8, 10, and 4 predicted activated upstream regulators in AD, KE, Li, and Pso, respectively (Table 1). In AD, the predicted activated upstream regulators (Z-scores) included *CNR1* (2.714), *BMP4* (2.431), *AMH* (2.181), and *CDK4/6* (2). In KE, predicted activated upstream regulators included *BMP4* (2.887), *CNR1* (2.496), *GH1* (2.433), *AMH* (2.385), *BMP2* (2.357), *TNFSF15* (2.213), *CCR2* (2.2), and *EDN1* (2.152). In Li, predicted activated upstream regulators included *BMP4* (2.942), *IL15* (2.449), *BMP2* (2.406), *Jnk* (2.397), *CNR1* (2.35), *IL7* (2.296), *CDK4/6* (2.236), *AMH* (2.181), *TNFRSF8* (2.164), and *Pkc(s)* (2.132). In Pso, predicted activated upstream regulators included *CNR1* (2.496), *BMP4* (2.3), and *CAV1* (2.216). Interestingly, *CNR1* and *BMP4* are the two common predicted activated upstream regulators. Consistent with the fact that the Rho GTPase family signalling is most significant, the most activated predicted upstream regulator in the two studies was *BMP4* (*p* = 1.01E−34), which can be regulated by Rho-kinase. Other important shared upstream regulators included *CNR1* (*p* = 6.43E−33), which is an important therapeutic target in inflammatory skin diseases^17^.

**Table 1:** Significant upstream regulators in inflammatory skin diseases.

	Upstream regulators	Molecules	Z-score	*P* value
AD	CNR1	GPCR	2.714	1.09E−05
	BMP4	Growth factor	2.431	6.23E−04
	AMH	Growth factor	2.181	1.10E−04
	CDK4/6	group	2	3.97E−02
KE	BMP4	Growth factor	2.887	2.15E−05
	CNR1	GPCR	2.496	2.64E−05
	GH1	Growth factor	2.433	7.32E−02
	AMH	Growth factor	2.385	6.30E−05
	BMP2	Growth factor	2.357	2.28E−04
	TNFSF15	Cytokine	2.213	2.45E−04
	CCR2	GPCR	2.2	1.48E−01
	EDN1	cytokine	2.152	7.70E−03
Li	BMP4	Growth factor	2.942	7.51E−06
	IL15	Cytokine	2.449	3.18E−03
	BMP2	Growth factor	2.406	2.76E−03
	Jnk	Group	2.397	7.08E−05
	CNR1	GPCR	2.35	4.40E−07
	IL7	Cytokine	2.296	2.58E−03
	CDK4/6	Group	2.236	3.04E−02
	AMH	Growth factor	2.181	4.53E−04
	TNFRSF8	Transmembrane receptor	2.164	1.38E−02
	Pkc(s)	group	2.132	8.48E−03
Pso	CNR1	GPCR	2.496	1.27E−04
	BMP4	Growth factor	2.3	4.16E−04
	CAV1	Transmembrane receptor	2.216	7.95E−02

AD; atopic dermatitis, KE; contact eczema, Li; lichen planus, Pso; psoriasis vulgaris, GPCR; G protein-coupled receptor.

### Identification of biomarkers in inflammatory skin diseases

Finally, we used IPA to search for DEGs that encoded biomarkers. We started by screening for genes that encoded biomarkers that had been described in any disease and performed IPA to identify the potential biomarkers of those DEGs. We found 416, 605, 556, and 722 biomarkers in AD, KE, Li, and Pso, respectively (Supplementary Table 5). For clinical use, biomarkers should be measurable in the blood; therefore, we sorted potential biomarkers that were measurable in blood samples, such as the extracellular biomarkers. This identified 44, 58, 50, and 75 extracellular biomarkers in each disease (Supplementary Table 6). Next, we checked whether these extracellular biomarkers were elevated in disease conditions compared to the levels in HC, which is indicated by a positive log fold-change value. We found 12 candidate common extracellular biomarkers for all inflammatory skin diseases as follows: ACR (acrosin), APOE (apolipoprotein E), ASIP (agouti signalling protein), CRISP1 (cysteine rich secretory protein 1), DKK1 (dickkopf WNT signalling pathway inhibitor 1), IL12B (interleukin 12B), IL9 (interleukin 9), MANF (mesencephalic astrocyte derived neurotrophic factor), MDK (midkine), NRTN (neurturin), PCSK5 (proprotein convertase subtilisin/kexin type 5), and VEGFC (vascular endothelial growth factor C) (Table 2). Among the 12 candidate common biomarkers, DKK1, IL12B, and VEGFC have been targeted by known drugs in clinical settings^18–20^. Moreover, the IL12B is a known biomarker for single-nucleotide polymorphisms in psoriasis, and a monoclonal antibody against the IL-12/23 subunit is used clinically^21,22^.

**Table 2:** Measurable common biomarkers in inflammatory skin diseases.

Symbol	Entrez gene name	AD	CE	LP	Pso	Average
ACR	Acrosin	0.279	0.579	0.412	0.632	0.476
APOE	Apolipoprotein E	0.824	1.427	0.566	0.539	0.839
ASIP	Agouti signaling protein	0.726	0.722	0.992	0.874	0.829
CRISP1	Cysteine rich secretory protein 1	0.39	0.226	0.189	0.464	0.317
DKK1	Dickkopf WNT Signaling Pathway Inhibitor 1	0.857	0.63	0.633	0.812	0.733
IL12B	Interleukin 12B	0.429	0.585	0.671	0.227	0.478
IL9	Interleukin 9	0.522	0.799	0.645	1.077	0.761
MANF	Mesencephalic astrocyte derived neurotrophic factor	0.624	0.755	0.696	0.434	0.627
MDK	Midkine	1.026	2.079	1.923	0.731	1.440
NRTN	Neurturin	0.616	1.444	1.535	1.685	1.320
PCSK5	Proprotein convertase subtilisin/kexin type 5	0.523	0.553	1.048	0.447	0.643
VEGFC	Vascular endothelial growth factor C	0.733	1.07	1.372	0.207	0.846

## Discussion

We found 238 shared DEGs that showed differing expressions between samples from inflammatory skin disease and HCs based on gene expression profiling data. Further bioinformatic analyses led to the identification of a wide variety of pathways and predicted upstream regulators. This complexity could explain the difficulties in identifying representative biomarkers. Although different types of biomarkers were found in each disease, we finally identified the following common biomarkers: ACR, APOE, ASIP, CRISP1, DKK1, IL12B, IL9, MANF, MDK, NRTN, PCSK5, and VEGFC, which are measurable in the serum and reflect extracellular biomarkers.

Previous studies by us and others have indicated altered expression of thousands of genes across multiple cell types locally and in blood samples from patients with inflammatory skin diseases^3–5^. This finding is consistent with the increasing recognition of the pathogenic complexity of inflammatory skin disease; however, the gene expression changes could ideally be exploited to identify highly accurate combinations of biomarkers^15^. From a clinical perspective, diagnostic proteins or soluble biomarkers in inflammatory skin disease are difficult to measure locally, within specimens from the diseased skin. Instead, analysis of blood samples would be more convenient. As mentioned in the introduction, there is a large collection of evidence about biomarkers or cytokine signatures in inflammatory skin diseases^6^. There is, however, a need for more accurate measurable biomarkers, which ideally should reflect the local disease activity. Furthermore, only few studies have tried to elucidate the inflammatory signatures and involved pathways as well as the upstream regulators in each disease, although all inflammatory skin diseases are similarly considered to be inflammatory autoimmune diseases predominantly driven by T cells. Therefore, in this study, we obtained a functional overview of the complex gene expression changes in different inflammatory diseases by focusing on pathways, upstream regulators, and measurable biomarkers rather than individual genes.

First, we checked pathways in each disease using DEGs identified from diseased patients versus HC, and comparative analysis showed quite similar patterns, which may explain the difficulties in the differential diagnosis of different inflammatory skin diseases. Despite the similar patterns observed in the comparative analysis, the rankings of individual significant pathways appeared different among each disease, reflecting the difficulty in biomarker prioritization. Next, we checked the predicted upstream regulators that affect DEGs and found that there are two common predicted activated upstream regulators—*CNR1* and *BMP4*. Sconocchia et al. demonstrated that BMP signalling plays an important role in inflammatory Treg-cell accumulation during skin inflammation^23^. Moreover, psoriatic lesions are marked by constitutive high BMP7/BMPR signalling in keratinocytes, which instructs inflammatory dendritic cells to enhance Treg-cell–stimulatory activity^23^. Moreover, Kim et al. showed that the BMP-4 expression of epithelial cells was higher in oral Li, which suggested that the overexpression of BMP-4 was a crucial factor for the apoptosis of epithelial cells in Li^24^. Furthermore, the human endogenous cannabinoid system (ECS) is a complex signalling network involved in a vast number of physiological processes. The endocannabinoids and cannabinoid receptor (CB) 1, which corresponds to *CNR1* gene signalling, may play a potent inhibitory role in human mast cell degranulation and activation in the airway mucosa and skin, suggesting that targeting the ECS in these tissues might well represent a novel strategy for the treatment of allergy. Martin-Fontecha et al. showed that mRNA expression level of *CNR1*, the gene encoding CB1 protein—the main component of the ECS—is upregulated in the tonsils and peripheral blood of patients with three different types of allergic diseases: allergic rhinitis, AD, and food allergy^25^. This previous finding reflected the fact that the predicted upstream regulator, *CNR1*, can be a potential therapeutic target that is mediated by the ECS. Collectively, our findings about the common predicted upstream regulators concur with the findings of previous studies.

Of note, the upstream regulators were predicted based on their known effects on the downstream groups of genes. Thus, if a group of genes showed coordinated changes, potential upstream regulators of those changes were identified based on previous experimental data that were accumulated in IPA^26^. However, for clinical purposes, the application of predicted upstream regulators based on gene expression profiles is impractical. Instead, a limited number of protein biomarkers that can be measured using routinely available methods, either in the blood or in local tissues, can be ideally used. Therefore, we ultimately checked whether extracellular biomarkers are elevated in disease states compared to the levels in HC, based on a positive log fold-change value. For all inflammatory skin diseases, we found 12 common biomarker candidates: ACR, APOE, ASIP, CRISP1, DKK1, IL12B, IL9, MANF, MDK, NRTN, PCSK5, and VEGFC. The relevance of ApoE, DKK1, IL12B, IL9, MANF, and VEGFC in specific inflammatory skin diseases was identified in several previous studies, as discussed further.

A meta-analysis of seven studies indicated that ApoE polymorphisms, especially the ε2 and ε3 alleles, are associated with an increased risk of psoriasis^27^. A genome-wide association study (GWAS) confirmed the association of the *IL12B* and *IL23R* genes with psoriasis^22^. Interestingly, robust IL12A and IL12B expression was found in patients with chronic Li, and the expression of *IL-9*, *IFN-γ*, and *IL-22* was higher in cutaneous Li^28^ than that in oral Li^29^. Polymorphisms in the *IL-9* and *IL-9R* genes have been associated with AD^30^, and the serum IL-9 levels were not only increased in AD patients compared with HC but were also positively correlated with the severity of AD^31^. Hui et. al proposed the expression of IL-9R in epidermal keratinocytes is increased by *IL-4*^32^; and *IL-9* was shown to induce gene expression and vascular endothelial growth factor (VEGF) secretion from mast cells by involving STAT-3 activation^33^. These aspects imply a pathophysiological role for IL-9/IL-9R in the skin of AD patients. Tej et al. found significantly higher levels of IL-9R in psoriatic skin than in the skin of HC, and an intradermal injection of IL-9 promoted *IL-17A* production; these findings suggest that IL-9 may play a role in the development of psoriatic lesions through Th17-associated inflammation and angiogenesis^34^. VEGF-C, a lymphangiogenesis marker in skin biopsies of psoriatic lesion^35^, was confirmed to be intensely expressed in Pso^36^ as well as in cutaneous Li^37^.

The treatment of ankylosing spondylitis and rheumatoid arthritis with TNF-α inhibitors was associated with a concurrent decrease in the DKK1 serum levels^38,39^. These abovementioned studies emphasize the role of DKK1 as a protagonist in chronic immune-mediated diseases; therefore, DKK1 may serve as a biomarker for the pathogenetic activity of these diseases. Owing to the significance of Wnt signalling in angiogenesis, Wnt antagonists, such as DKK1, have been considered as potential treatments for neovascularization-related disorders^40^. Gudjonsson et al. reported evidence of altered Wnt signalling in the psoriatic skin^41^; therefore, DKK1, a Wnt antagonist, can be a possible biomarker in inflammatory skin disease. The bioinformatics analysis of the proteome from the serum of AD patients demonstrated the altered landscape of immunological aberrations, and MANF was significantly increased in AD patients compared to HC^42^. These findings support the assumption that DKK1 and MANF are potential biomarkers despite the protein–gene gap.

Recent findings have established the skin as a peripheral neuroendocrine organ that is tightly networked to the central stress axes^43–47^. Specifically, epidermal and dermal cells produce, and respond to, classical stress neurotransmitters, neuropeptides, and hormones. This production is modified by ultraviolet radiation and biological, chemical, and physical factors^43,44^. Examples of potent epidermal products include biogenic amines (catecholamines, serotonin, and *N*-acetyl-serotonin), acetylcholine, melatonin and its metabolites, proopiomelanocortin-derived ACTH, β-endorphin and MSH peptides, corticotropin-releasing factor and related urocortins, corticosteroids and their precursor molecules, thyroid-related hormones, opioids and cannabinoids^45,46^. The production of these molecules in the skin is hierarchical, following the algorithms of classical neuroendocrine axes (e.g., hypothalamic pituitary adrenal axis, hypothalamic-thyroid axis, serotoninergic/melatoninergic, catecholaminergic and cholinergic systems). The deregulation of these systems may be involved in the etiology of skin diseases, and the control of these systems constitute novel targets through the use of specific agonists or antagonists, especially for therapy of skin diseases^45–47^. In this study, we found that 238 DEGs partially overlapped between different inflammatory skin diseases and identified 12 candidate biomarkers that overlapped, namely, ACR, APOE, ASIP, CRISP1, DKK1, IL12B, IL9, MANF, MDK, NRTN, PCSK5, and VEGFC. In the future, biomarkers might play a central role in personalized therapy by facilitating the identification of patients who will not respond to a certain treatment or those who might get adverse reactions^48^. Moreover, biomarkers play a very important role in deepening our understanding of the pathogenesis of psoriasis and could facilitate the development of biological therapies^49^. As mentioned earlier, the pathogenic and diagnostic relevance of these biomarkers is supported by not only single-nucleotide polymorphism and GWAS studies, but also through studies of the mechanism, such as Wnt signalling^50^. When considered with our findings, future studies are warranted to evaluate ACR, APOE, ASIP, CRISP1, DKK1, IL12B, IL9, MANF, MDK, NRTN, PCSK5, and VEGFC as candidate biomarkers in inflammatory skin diseases.

The limitations of this study include the fact that only one microarray was analysed, and that mRNA levels may not necessarily correspond to the protein expression levels. Nonetheless, the study findings are supported by those from previous studies of individual mechanisms in each inflammatory skin disease. Here, we present a systems-level overview of pathways and upstream regulators, which indicates the complexity of pathogenesis and, potentially, the relative importance of the identified mechanisms. This systems-level analyses on bulk microarray data should be assessed with data from cutting edge technologies such as single-cell RNA sequencing for reproducibility of potential biomarkers. Next, pathway analyses can be confounded by knowledge bias, inaccurate knowledge of gene interactions, or how gene interactions vary in different cell types. Despite this limitation, our pathway analyses were supported by the partially consistent results across different studies, and the findings are being agreement with the current understanding of disease mechanisms in inflammatory skin disease.

Collectively, the current system-level analysis based on profiling microarray data suggested possible combinatorial biomarkers for inflammatory skin diseases. Future studies for the identification of combinatorial biomarkers are warranted.

## Methods

### Identification and selection of eligible gene expression dataset

We systematically mined the Gene Expression Omnibus (GEO) database^51^ for expression profiling datasets. The following key words and their combinations were used: ‘inflammatory skin disease’, ‘human’, ‘microarray’, ‘gene expression dataset’, ‘tissues’, and ‘biopsy’. Specifically, the gene expression data were extracted for each diseased condition in comparison to the data of HCs. The inclusion criteria were specified and strictly followed for dataset selection: human case/control study, comparable conditions, untreated samples, and the availability of raw and processed data. In this study, we finally selected GSE 63,741 because it included data on diseased skin tissues, as well as control skin tissues, and it had enough number of patients, covering four different kinds of inflammatory skin diseases based on the same microarray platform to decrease possible technical bias.

### Analysis of gene expression data

We first identified DEGs between inflammatory skin diseases and HCs using GEO2R^53^. The data were annotated using the National Centre for Biotechnology Information-generated platform and adjusted for multiple testing using the Benjamini–Hochberg procedure^16^. The data were then sorted only to include significant DEGs (*q* value < 0.05 based on false discovery rate) for downstream analysis^16^.

### Identification of DEGs

For bulk microarray data, DEGs were identified using the LIMMA R package (Bioconductor, version 3.5) as described in the ‘Analysis of gene expression data’ that were reported in the Methods section of previous studies^16,54,55^. A negative binomial distribution was used to define the dataset with the lowest detection limit of 0.5. Genes were considered as significant DEGs if the adjusted *p* value was less than 0.05.

### Ingenuity pathway analysis

Our bioinformatics strategy was based on finding pathways among the DEGs, and upstream regulators of the DEGs. The objective of pathway analysis is the obtain an overview of disease-associated mechanisms, while the objective of upstream regulators is to find key regulators of such mechanisms. The analyses were performed using the IPA software (Qiagen, Hilden, Germany)^26^. The IPA includes a global network, which is based on the manual curation of a vast body of medical literature and biomedical databases and is continuously updated^56^. The core analysis in IPA was used to identify pathways that were significantly enriched among the DEGs, as well as to predict upstream regulators of those DEGs, which were either activated or inhibited. The statistical analysis was performed using Fisher’s exact test (right-tailed) within the IPA software^26^. We performed an analysis of IPA’s upstream regulators to identify transcription factors that were predicted to be activated based on the activation Z-score^26^. The upstream regulators of groups of interacting DEGs were identified by Upstream Regulator Analysis (URA) that is based on the Ingenuity Knowledge Base database. For the upstream regulator analysis, we focused on seven molecule types—specifically, cytokines, complexes, groups, growth factors, G-protein-coupled receptors, ligand-dependent nuclear receptors, and transmembrane receptors^16^. IPA has a biomarker filter function, which identifies the most promising and relevant biomarker candidates in experimental data. Using the function of biomarker filter, we prioritized molecular biomarker candidates based on key biological characteristics and selected extracellular biomarkers that could be detected in blood.

### Protein–protein network interaction

We used Cytoscape software to visualize protein–protein interaction network for common DEGs. Cytoscape is an open-source software platform for visualizing molecular interaction networks and biological pathways and integrating these networks with annotations, gene expression profiles and other state data.

### Ethics declaration

This study was conducted in accordance with the Declaration of Helsinki. Using GEO2R, we identified DEGs between lesional and HC skin samples from patients with AD, KE, Li, and Pso. Skin samples of 150 donors (30 patients with AD, KE, Li, Pso, and HC, respectively) were analysed in GSE 63741. Only biopsies taken from untreated patients with typical skin lesions were included. The patients with KE included those with allergic contact dermatitis and irritant contact dermatitis. The public GEO data which can be assessable for everyone was annotated using the national centre for biotechnology information (NCBI) generated platform.

## Supplementary Information

Supplementary Information 1.

Supplementary Information 2.

Supplementary Information 3.

Supplementary Information 4.

Supplementary Information 5.

Supplementary Information 6.

Supplementary Information 7.

Supplementary Information 8.

Supplementary Information 9.

## Data Availability

The processed bulk data generated in this study are publicly available on GEO database (https://www.ncbi.nlm.nih.gov/geo/) with accession no. GSE63741.
