# Supplementary figures and images for: Integrated bioinformatic analysis of gene expression profiling data to identify combinatorial biomarkers in inflammatory skin disease
Source: Sci Rep. 2022 Apr 7;12:5889. doi: 10.1038/s41598-022-09840-3 (PMC8989986; doi:10.1038/s41598-022-09840-3)

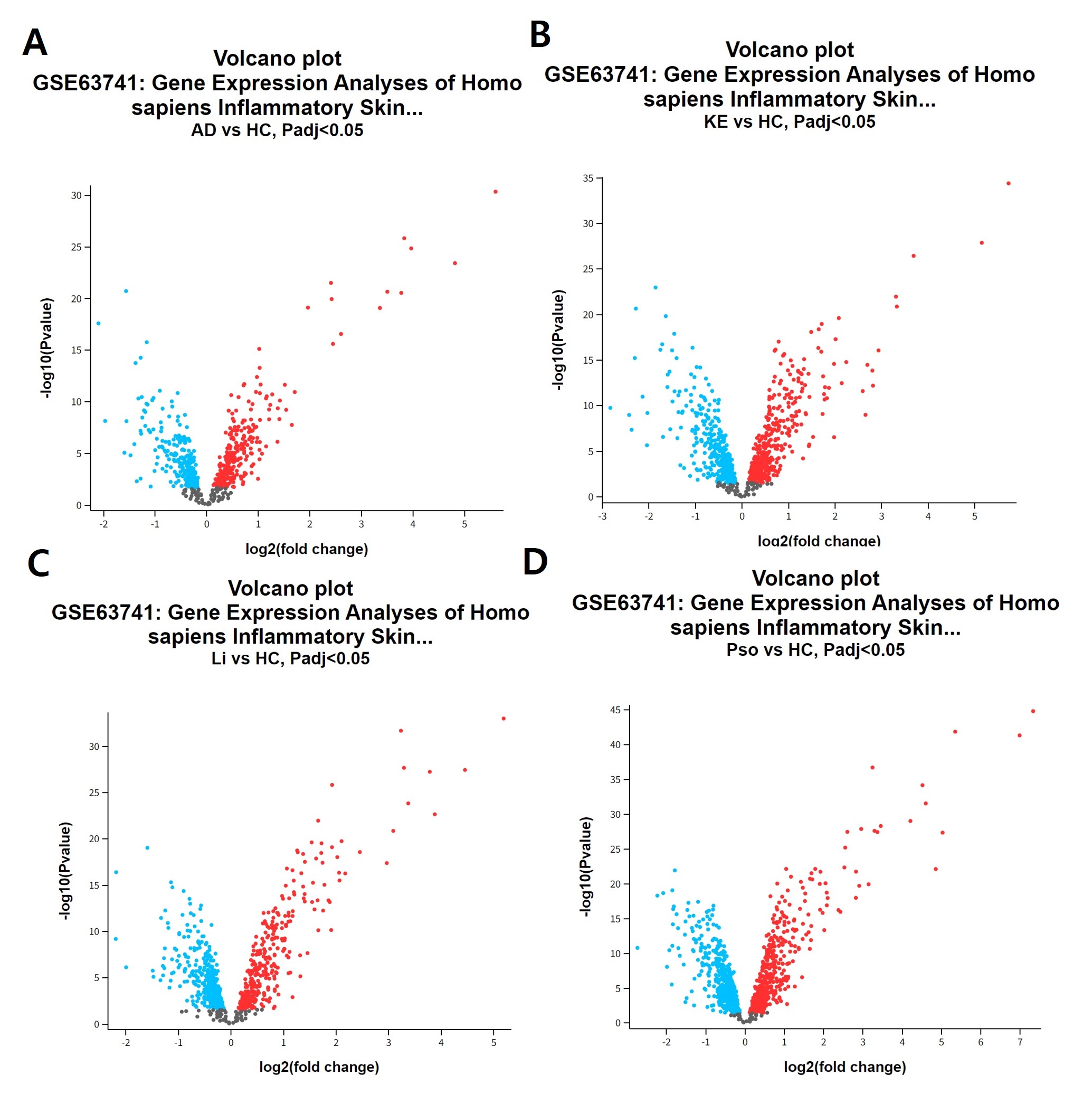

Supplement: Supplementary file 1 — Supplementary Information 1. [file 41598_2022_9840_MOESM1_ESM.jpg]

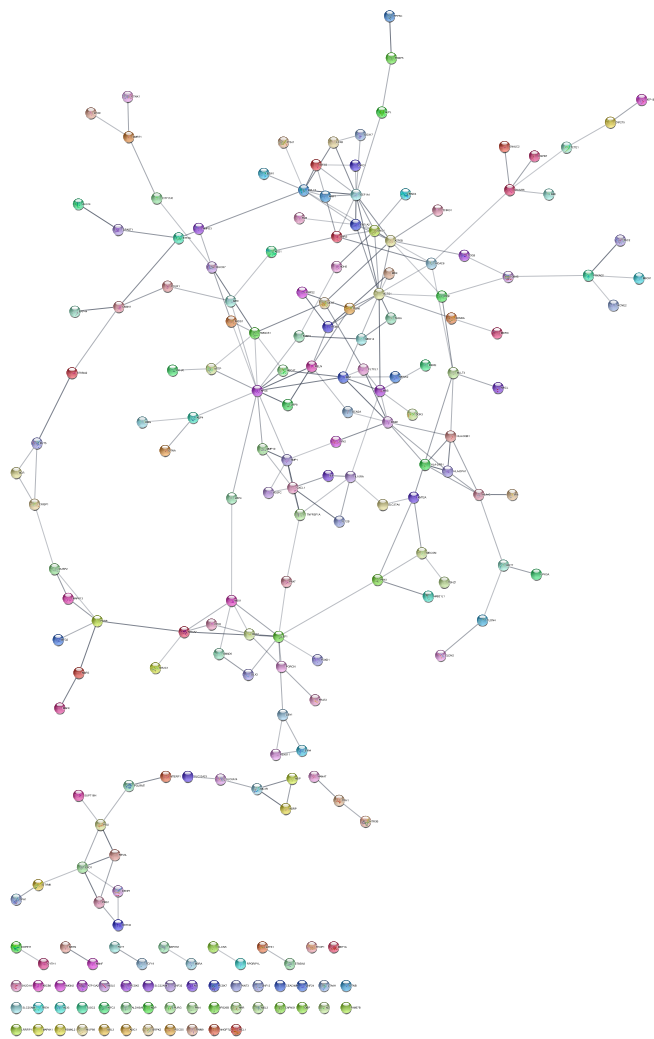

Supplement: Supplementary file 3 — Supplementary Information 3. [file 41598_2022_9840_MOESM3_ESM.pdf]
