# Supplementary material for: Integrated bioinformatic analysis of gene expression profiling data to identify combinatorial biomarkers in inflammatory skin disease
Source: Sci Rep. 2022 Apr 7;12:5889. doi: 10.1038/s41598-022-09840-3 (PMC8989986; doi:10.1038/s41598-022-09840-3)
Supplement: Supplementary file 9 — Supplementary Information 9. [file 41598_2022_9840_MOESM9_ESM.docx]

**RESEARCH ARTICLE**

**Integrated bioinformatic analysis of gene expression profiling data to identify combinatorial biomarkers in inflammatory skin disease**

Heejin Bang^1^, Ja Eun Kim^2^, Hyun Su Lee^3^, Sang Man Park^3^, Dong-Joon Park^3^, Eun Jung Lee^3*^

^1^Department of Pathology, Konkuk University Medical Center, Konkuk University School of Medicine, Seoul, Republic of Korea

^2^Yonsei University Wonju College of Medicine, Wonju, Republic of Korea

^3^Department of Otorhinolaryngology, Yonsei University Wonju College of Medicine, Wonju, Republic of Korea

**Additional files**

Additional file 1: Supplementary Figure 1. Volcano plot of differentially expressed genes in each inflammatory skin disease. (A) Atopic dermatitis versus healthy control, (B) Contact eczema versus healthy control, (C) Lichen planus versus healthy control, (D) Psoriasis vulgaris versus healthy control (blue dot; downregulated genes, red dot; upregulated genes, gray dot; not significant genes).

Additional file 2: Supplementary Table 1. Identification of differentially expressed genes in skin biopsies from patients with inflammatory skin disease

Additional file 3: Supplementary Figure 2. Protein-protein interaction network from common differentially expressed genes from four kinds of inflammatory skin diseases

Additional file 4: Supplementary Table 2. Integration of gene ontology terms for common differentially expressed genes from four kinds of inflammatory skin diseases

Additional file 5: Supplementary Table 3. Identification of pathways in skin biopsies from patients with inflammatory skin disease

Additional file 6: Supplementary Table 4. Identification of upstream regulators in skin biopsies from patients with inflammatory skin disease

Additional file 7: Supplementary Table 5. Identification of biomarkers in skin biopsies from patients with inflammatory skin disease

Additional file 8: Supplementary Table 6. Identification of extracellular biomarkers in skin biopsies from patients with inflammatory skin disease

*Detail information is described in the first tab of supplementary excel files.
